# Neglected Tropical Diseases and the 2012 US Presidential Election

**DOI:** 10.1371/journal.pntd.0001431

**Published:** 2011-11-29

**Authors:** Peter J. Hotez, Karen A. Goraleski

**Affiliations:** 1 National School of Tropical Medicine and Department of Pediatrics and Molecular Virology & Microbiology at Baylor College of Medicine, Houston, Texas, United States of America; 2 Sabin Vaccine Institute and Texas Children's Hospital Center for Vaccine Development, Houston, Texas, United States of America; 3 American Society of Tropical Medicine and Hygiene, Deerfield, Illinois, United States of America

*Within the United States government both Democrats and Republicans have championed support to control and eliminate neglected tropical diseases. In the next year leading up to the US presidential and congressional elections, the global NTD community has an important opportunity to present the case for NTD funding to remain as an essential component of US international development assistance.*

The neglected tropical diseases (NTDs), a group of 17 poverty-promoting chronic parasitic and related infections, such as hookworm and other intestinal helminthiases, schistosomiasis, lymphatic filariasis, onchocerciasis, and trachoma, represent the most common infections of the world's poor [1], [2]. Today, the United States government is the largest supporter of NTD control and elimination efforts worldwide, especially with respect to administering rapid impact packages of NTD drugs in national control programs in Africa and Asia [3], [4].

It is not uncommon for those in academia and non-profit settings (in the US and abroad) to assume that Democratic members of Congress provide much of the leadership for sustaining the US commitment to NTD control. Presumably, these assumptions are based on the Democratic Party's historic commitment to social programs and international development assistance. However, NTD control efforts have a proven track record of bipartisan presidential and congressional support.

Democratic President Jimmy Carter (1977–1981) was the first US president to widely acknowledge and promote the control, elimination, or eradication of onchocerciasis, guinea worm, and other NTDs in the years following his term in office [5]. Republican President George W. Bush (2001–2009), who is appropriately lauded for the establishment of the President's Emergency Plan for AIDS Relief (PEPFAR), is also the first US president to incorporate integrated NTD control into the US government's broader global health agenda. Indeed, President Bush was the first US president to champion rapid impact packages of drugs for the NTDs and the scale up of national control programs in sub-Saharan Africa and elsewhere, and pledged US$350 million toward NTD treatment over a 5-year period (FY 2009–FY 2013) [6].

Democratic President Barack Obama has expanded NTD control and elimination programs through integrated control [4] and his Global Health Initiative, while leading efforts to maintain NTD funding levels even in the face of enormous pressure within the US Congress to significantly cut the international affairs budget. Yet, the US is still only half way in reaching the US$350 million pledge made in 2008 to fight NTDs. Both former Democratic President William J. Clinton (1993–2001) and US Secretary of State Hilary Clinton remain knowledgeable and enthusiastic supporters of NTD control and elimination, and the Global Network for Neglected Tropical Diseases was launched at the Clinton Global Initiative in 2006 [1].

Similarly, US Congressional support today for NTDs continues to cross party lines. Members of the Senate, such as the late Senator Edward Kennedy (D-Massachusetts), former Senator Sam Brownback (R- Kansas), and current Senators Sherrod Brown (D-Ohio) and Patrick Leahy (D- Vermont) rank among the most ardent champions on this issue, while in the House of Representatives NTDs were successfully added to the bipartisan Congressional Malaria Caucus, creating a new Malaria and NTD Caucus in 2009 [7]. Former Secretary of Health and Human Services and Wisconsin Governor Tommy Thompson (R-Wisconsin) also stands out as an ardent supporter of NTD control [8]. Therefore, it is fair to say that support to control and eliminate NTDs represents a truly bipartisan effort.

To fully appreciate the current level of bipartisan support, it is important to acknowledge the years of purposeful and strategic engagement by the NTD science and advocacy communities. We would not be where we are today without the individual and collective efforts of researchers, program professionals, and other advocates who have petitioned members of Congress to maintain and expand NTD funding as part of a comprehensive global health and development policy. We must continue advocating aggressively for support for tropical medicine at the National Institutes of Health, the US military laboratories both at the Walter Reed Army Institute of Research and overseas, and for the NTD Program of the United States Agency for International Development. As part of our advocacy outreach it is also worth highlighting the hidden burden of tropical diseases that occur in areas of poverty in the US, but especially in Texas and the Gulf Coast [8], as well as the potential global power of NTD control when it is effectively incorporated into US foreign policy [9].

How will this hard-fought bipartisanship fare in the November 2012 US presidential election? The Obama Administration has a clear track record of supporting NTD control efforts. If elected to a second term, there is every reason to anticipate that Obama's support for NTD control and elimination will continue, barring a worsening of the US economic outlook. Political history also shows us that we should not dismiss the Republican Party's potential to maintain NTDs as part of their foreign policy agenda, particularly given the potential link between poverty, often compounded by NTDs, and terrorism [9]. Although the Republican presidential race is still taking shape, and being defined by domestic issues, we can expect that global affairs will become an important element to the presidential race. Even the most fiscally conservative Republicans may be expected to champion control of the NTDs (sometimes referred to as the “Biblical diseases” because of descriptions of these conditions in ancient texts [10]) given the cost-effectiveness of NTD programs and their potential to help reduce poverty levels in emerging economies.

This election cycle offers us an important opportunity to meet and brief the Republican presidential candidates about NTDs. Candidates are—more than ever—eager to meet with as many audiences as possible and learn their specific issues, including this one.

There is no doubt that a US government commitment to NTD control absolutely depends on maintaining bipartisan support. Similarly, bipartisan support depends on the continued efforts of each of us to offer our time and expertise to members of the US Congress and their staff to enable them to make the medical, socioeconomic, and national security case for adequate funding and policies for NTDs. We all have a special obligation in ensuring that the NTDs are not neglected even further. Gaps in funding still exist, and engaging both Congressional Democrats and Republicans on NTDs must remain a top priority for each of us in the science community. Your research and program funding depends on it; US evidenced-based policies depend on it; and, millions of people around the world who live in poverty depend on it as well.
